# Laboratory evaluation of the efficacy of lotilaner (Credelio™) against *Amblyomma cajennense* (*sensu lato*) infestations of dogs

**DOI:** 10.1186/s13071-018-3116-x

**Published:** 2018-10-03

**Authors:** Pedro Veloso Facury Lasmar, Martin Murphy, Steve Nanchen, Jason Drake, Katherina Coumendouros, Debora Azevedo Borges, Priscila Cardim de Oliveira, Fábio Barbour Scott

**Affiliations:** 1 Elanco Animal Health, Av. Morumbi, 8264, São Paulo, SP 04703–002 Brazil; 2 Elanco Animal Health, Mattenstrasse 24a, CH-4058 Basel, Switzerland; 3 Elanco Animal Health, 2500 Innovation Way, Greenfield, IN 46140 USA; 4 Rural Federal University of Rio de Janeiro, BR 465 km 7 UFRRJ, Seropédica, RJ Brazil

**Keywords:** Canine, Ticks, Isoxazoline, Effectiveness, Cayenne tick

## Abstract

**Background:**

The ixodid tick *Amblyomma cajennense* (*sensu lato*) complex, widespread throughout South and Central America, is also present in Mexico, Texas and Florida. As a vector of *Rickettsia rickettsii*, and potentially of other pathogens, infestations with *A. cajennense* present a substantial health risk to humans, dogs and other mammals. Oral administration of lotilaner flavored chewable tablets (Credelio^TM^, Elanco) to dogs was previously shown to rapidly provide killing activity of infesting ticks. This study investigated lotilaner’s efficacy against *A. cajennense* (*s.l*.).

**Methods:**

Twenty purpose-bred Beagles (10 male and 10 female) were ranked by Day -5 burdens of nymphal *A. cajennense* (*s.l*.) and randomized to either treatment with lotilaner or to a sham-treated control group. On Day 0, dogs were fed within approximately 30 min prior to oral lotilaner administration at as close as possible to 20 mg/kg, the minimum dose rate. For efficacy assessments, tick counts were completed 48 h post-treatment or 48 h after experimental challenge infestations with 200 nymphal *A. cajennense* (*s.l*.) on Days -7, -2, 7, 14, 21 and 28.

**Results:**

Tick infestations in the control group dogs ranged from a low of 43 to 95, with the average infestation remaining above 25% at each assessment, thereby meeting the requirement for efficacy comparison with the treated group. Lotilaner efficacy was 100% within 48 h post-treatment, and at nine days post-treatment. Efficacy was greater than 99% at all subsequent assessments through Day 30. No treatment-related adverse events were observed.

**Conclusion:**

The results demonstrate that lotilaner, administered orally to dogs at a minimum dose of 20 mg/kg is well tolerated, provides rapid reduction of existing *A. cajennense* (*s.l*.) tick infestations, and provides sustained residual protection for at least 30 days against subsequent infestation by *A. cajennense* (*s.l*.).

## Background

The ixodid tick *Amblyomma cajennense* (*s.l*.) is important as a vector of *Rickettsia rickettsii*, the cause of Brazilian/ Rocky Mountain spotted fever. This is a disease of dogs and humans which is potentially fatal, particularly in Brazil where the strain of *R. rickettsii* is reported to be of greater pathogenicity than in the United States [1–4]. *Amblyomma cajennense* species complex is widely distributed throughout tropical and sub-tropical regions of South and Central America, is present in Mexico, Texas and Florida, and on the Caribbean islands including Cuba [3–10]. In Brazil, capybaras act as the primary host of *A. cajennense* (*s.l*.) and most endemic areas of *R. ricketsii* infections are those with large, free living populations of this rodent [3, 4]. While dogs are not seen as being a primary host for *A. cajennense* (*s.l*.), nymphal stages are less host specific than the adult stage and are reported to be aggressive feeders on humans and dogs [11, 12]. As such, infestations with *A*. *cajennense* (*s.l*.) present a significant risk to human and canine health across a broad geography [1–6]. Ectoparasiticides introduced to treat and control canine tick infestations in affected areas should be shown to be effective against this tick species.

Targeting ligand-gated GABA receptors, the isoxazolines compounds have a novel mode of action, distinct from earlier ectoparasiticidal treatments, that causes paralysis and death of fleas and ticks [13, 14]. Building on the efficacy against fleas and tick of the first isoxazoline, afoxolaner, which was first approved in 2013, subsequent members of the family that have become available are fluralaner and sarolaner, first approved in 2014 and 2016, respectively, and most recently, lotilaner, which was first approved in 2017. Following oral administration under fed conditions, peak lotilaner concentrations are reached within two hours, with a corresponding rapid onset of activity against fleas and ticks [15–17]. Lotilaner efficacy has been demonstrated against a number of tick species including, but not limited to, *Rhipicephalus sanguineus* (*sensu lato*), *Dermacentor reticulatus*, *Dermacentor variabilis*, *Ixodes scapularis*, *Ixodes ricinus* and *Amblyomma americanum* [18, 19]. With a half-life of 30 days, lotilaner has been shown to provide at least a month of activity against infesting ticks [15, 17–19]. To further expand the understanding of the activity of lotilaner flavored chewable tablets against ticks, a study was initiated to investigate efficacy in eliminating existing burdens of *A. cajennense* (*s.l*.) and in maintaining efficacy against challenge through the month following oral administration at a minimum dose rate of 20 mg/kg.

## Methods

The study was designed and completed during 2015 in accordance with the second edition guidelines from the World Association for the Advancement of Veterinary Parasitology (WAAVP) for evaluating the efficacy of parasiticides for the treatment, prevention and control of flea and tick infestations of dogs and cats and with current local regulatory standards [20]. According to Labruna & Pereira [10], the highest prevalence of infestation of *A. cajennense* (*s.l*.) in domestic dogs in Brazil occurs during its larval and nymphal stages. Infestations with adult *A. cajennense* (*s.l*.) ticks is less prevalent in dogs [10]. In addition, the attachment rate of adult ticks of this species in laboratory dogs is very low, not allowing for the minimum attachment rate of 25% recommended by international guidelines [10, 12]. For these reasons, nymphal stages were used for infestation of dogs in this study.

### Animals and housing

**Fig. 1:**
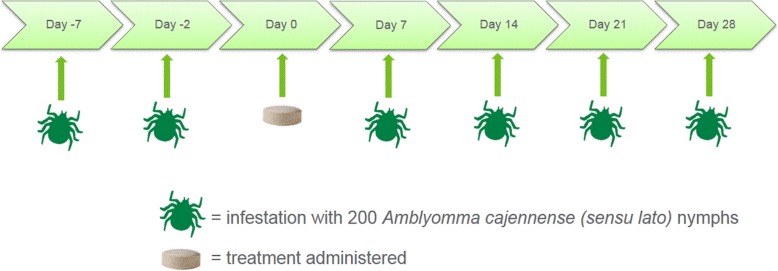
Timings of infestations and treatment

Twenty-six purpose-bred Beagle dogs (male and female) were acclimatized, and of these 20 (10 male and 10 female) were retained in the study. Each dog was identified with a subcutaneous microchip identification number. A dog was eligible to be enrolled in the study if it was at least six months of age and 6.8 kg on Day -7, clinically healthy with no pre-existing conditions (e.g. injury, trauma, disease) that could have affected the study, and if it sustained a pre-treatment tick infestation of at least 50 ticks (25% of the infesting dose) (Fig. 1). Dogs were excluded if pregnant or lactating, or if treated with an ectoparasiticide in the eight weeks preceding selection for the study (or within six months for isoxazoline compounds). For the study, dogs were housed individually in concrete-floored pens measuring approximately 1.5 × 1.5 m and for socialization and exercise were placed into a fenced corridor daily, except on days when tick infestations were completed. For socialization, dogs within the same treatment group were separated by sex and socialized together, with separate designated areas for each treatment group to avoid any risk of between-group cross-contamination. Animals remained in the same socialization group throughout the study. At least one toy/chew was made available at all times to each dog when housed individually. Dogs were fed a commercially available high quality complete canine dry food diet except on Day 0, when canned food was offered to all dogs, mixed with their dry ration to encourage consumption. Water was provided *ad libitum*.

### Randomization and treatments

Within sex, dogs that met all inclusion and exclusion criteria, including at least a 25% tick attachment rate, were ranked in order of descending Day -5 tick count. The 10 dogs of each sex with the highest tick counts were then included in the randomization process. The first two animals of each sex were allocated to form a block, the next two a second block, and so on. Dogs within each block were assigned, using random order numbers, to either treatment with lotilaner or to be a sham-treated control. Within approximately 30 min before dosing, all dogs had consumed a partial daily food ration.

Lotilaner was administered orally (whole tablets) at as close as possible to the targeted minimum dose rate of 20 mg/kg. Each dose was calculated based on bodyweight, to the nearest 0.1 kg, measured on Day -2. Control group dogs were sham-dosed on Day 0 using a process that matched the handling of the lotilaner-treated dogs, including removing the dog from its individual run and opening and massaging the dog’s throat.

### Tick infestations and counts

Ticks had been field-collected from horses located in the state of Rio de Janeiro in the southeast region of Brazil during 2015 and were subsequently maintained in the Laboratory of Experimental Chemotherapy in Veterinary Parasitology of the Federal Rural University of Rio de Janeiro, Brazil. The nymphal stage of *A. cajennense* (*s.l*.) was used for the study challenges because the highest prevalence of infestation of *A. cajennense* (*s.l*.) in domestic dogs in Brazil occurs during its larval and nymphal stages, while infestations with adult ticks is less prevalent [10]. In addition, the attachment rate of adult ticks of this species in laboratory dogs is very low, not allowing for the minimum attachment rate of 25% recommended by international guidelines. Each dog was experimentally infested in its pen with 200 viable unfed nymphs on Days -7, -2, 7, 14, 21 and 28 (Fig. 1). Dogs were not sedated for either tick application or counting. The ticks were applied gently to the dorsal rump area and allowed to crawl into the hair coat to select an attachment site. Ticks applied on Day -7 were counted and removed on Day -5, 48 (± 2) h after the first infestation. Ticks applied on Day -2 were counted and removed on Day 2, 48 (± 2) h after lotilaner administration. Since Day 2 tick counts were 4 days after initial infestation, the kennel floors were also inspected for engorged ticks since nymphal ticks may complete feeding in 3–4 days. No engorged nymphs were found on kennel floors. On Days 9, 16, 23 and 30 the ticks were counted and removed, 48 (± 2) h post-infestation. For counting ticks on treatment and control group dogs, each dog’s hair was pushed manually against its natural lie so that the skin and ticks were visible. A systematic examination for ticks included exploration of each dog’s head, limbs and all dorsal and ventral areas.

### Assessment of efficacy

Efficacy was determined by the reductions in live, attached tick counts on lotilaner-treated dogs, relative to control dogs, 48 h after treatment on Study Day 0 and 48 h after each subsequent infestation on Days 9, 16, 23 and 30. Arithmetic and geometric mean group efficacies were calculated according to Abbott’s formula as follows:

$$ \mathrm{Efficacy}\ \left(\%\right)=100\times \left(\mathrm{Mc}-\mathrm{Mt}\right)/\mathrm{Mc} $$

where Mc is the untreated control group’s mean number of live ticks on dogs, and Mt is the treated group’s mean number of live ticks on dogs. The Shapiro Wilk test was used to evaluate data normality. As this showed substantial deviation from normality, the Mann-Whitney test was utilized to compare the mean values between the live nymph counts of the groups. Calculation of geometric means was performed using logarithm transformed counts (count +1) with one (1) subtracted, subsequently, from the result. All analyses were performed using SAS version 9.2 software. Lotilaner was considered effective if the average live tick attachment rate in the control group was at least 25%, if there was a statistically significant difference (*P* < 0.05) between the treated group and the control group, and if the treated group had a calculated efficacy of at least 90%.

## Results and discussion

The doses of lotilaner administered to study dogs ranged from 20.3–25.0 mg/kg, which was as close to 20 mg/kg as possible using commercially available tablets without dropping below the minimum approved dose of 20 mg/kg. There were no treatment-related adverse events. Tick infestations in the control dogs ranged from a low of 43 up to a maximum of 95, with the average infestation rate remaining above 25% at each assessment, thereby meeting the requirement for adequate infestation and efficacy comparison with the treated group (Table 1).

**Table 1:** Summary and efficacy of live attached tick counts 48 hours post-treatment and following post-treatment infestations

		Day of tick count					
		-5	2	9	16	23	30
Control	Arithmetic mean ± SD	60.6 ± 7.1	60.0 ± 7.0	62.4 ± 13.7	62.0 ± 12.7	62.1 ± 14.8	63.2 ± 12.5
	Geometric mean	60.2	59.6	61.2	60.9	60.7	62.1
Lotilaner	Arithmetic mean ± SD	61.1 ± 9.7	0.0 ± 0.0	0.0 ± 0.0	0.3 ± 0.9	0.4 ± 0.8	0.6 ± 1.1
	Efficacy (%)	na	100	100	99.5	99.4	99.1
	Geometric mean	60.5	0.0	0.0	0.1	0.2	0.4
	Efficacy (%)	na	100	100	99.8	99.6	99.4
Comparison		*Z* = 0.11, *P* = 0.8951	*Z* = 4.00, *P* <0.0001	*Z* = 4.00, *P* <0.0001	*Z* = 3.92, *P* <0.0001	*Z* = 3.87, *P* <0.0001	*Z* = 3.82, *P* <0.0001

*Abbreviations*: SD, standard deviation; na, not applicable

**Fig. 2:**
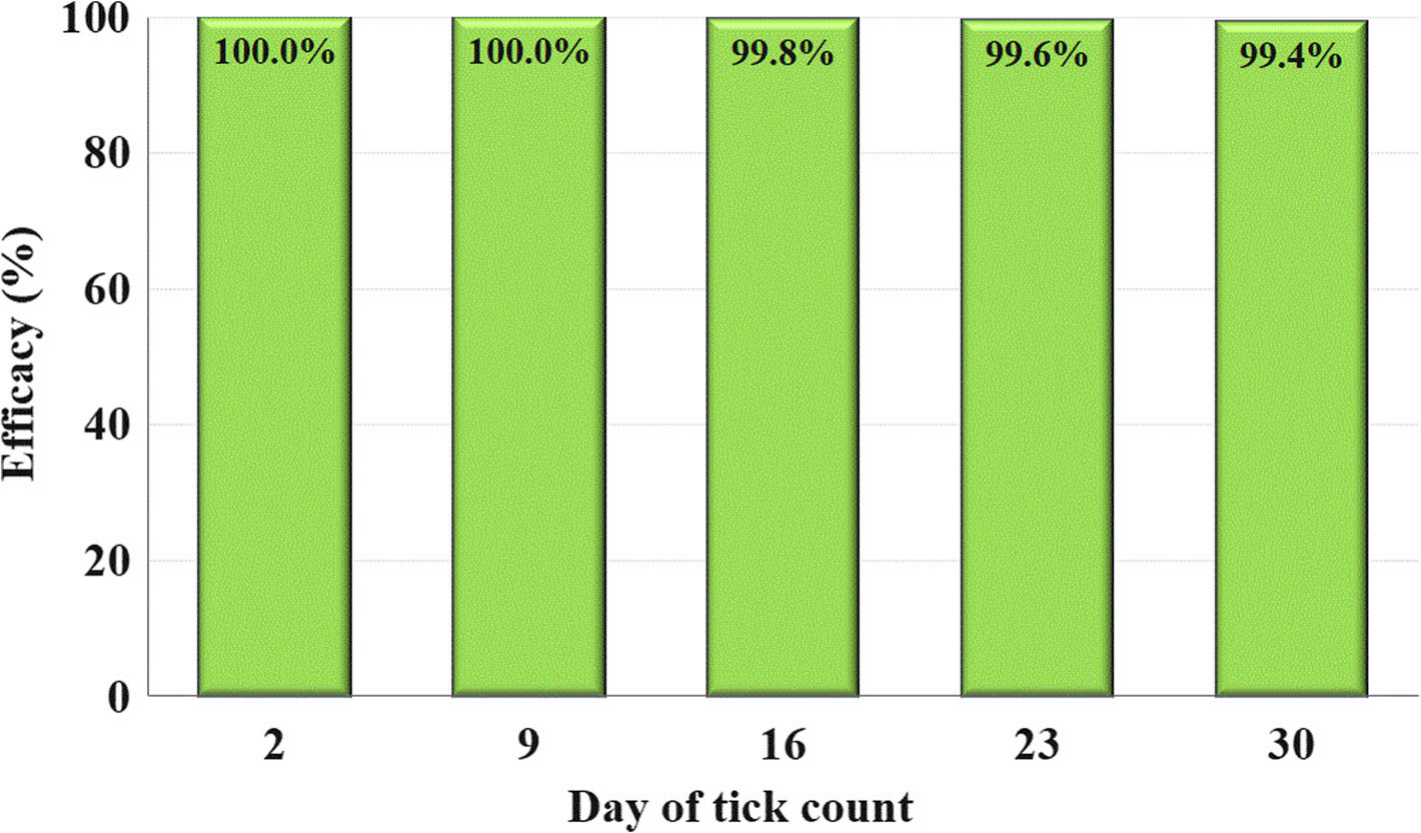
Percent efficacy in geometric mean *A. cajennense* tick counts of lotilaner-treated dogs compared to untreated control group dogs

Within 48 h post-treatment, no live ticks were found attached to any lotilaner-group dogs, and efficacy remained at, or close to 100% at every post-treatment assessment (Table 1, Fig. 2). From each infesting dose of 200 ticks, the maximum number of live ticks found on any lotilaner-group dog in the post-treatment period was three. Only one dog was infested on Day 16 (three ticks), two dogs on Day 23 (each with two ticks) and three on Day 30 (with one, two and three ticks). Between-group differences in mean counts of live *A. cajennense* (*s.l*.) were significant (*P* = 0.0002) at every post-treatment assessment.

This is the second report describing the efficacy of a treatment against *A. cajennense* (*s.l*.) infestations in dogs. The first such report described a study that used a similar protocol to that of our study to investigate the efficacy of sarolaner [21]. That study produced 100% efficacy for sarolaner by 48 h following Day 12 infestation and ≥ 99.6% by 48 h following Day 33 infestation, similar to those that we report for lotilaner [21]. While the results of the two studies suggest that efficacy against *A. cajennense* (*s.l*.) may be an isoxazoline class effect, other studies suggest that different members of the class do not necessarily provide the same degree of efficacy, particularly against *Amblyomma* spp. For instance, there are only two reports of afoxolaner efficacy against *A. americanum* - in one of these at 24 h post-infestation afoxolaner efficacy was less than 80% from Day 14 through Day 30 while under the same conditions sarolaner efficacy remained at greater than 90% until Day 28 [22]. Studies assessing lotilaner efficacy against *A. americanum* at 48 h post-treatment and post infestation have demonstrated efficacy of 100% throughout, except at nine days post-treatment when efficacy was 99% [19].

For lotilaner, the results of this study align with earlier reports of efficacy against ticks in laboratory and field studies. One study demonstrated efficacy of 70% against existing infestations with *I. ricinus* within four hours post-treatment [17]. A report of a series of studies involving experimental infestations with commonly occurring European ticks showed that lotilaner efficacy remained at least 95% through 35 days post-treatment and a field study across three European countries subsequently confirmed lotilaner effectiveness against field challenge [18, 23]. Similar efficacy was reported following induced infestations of commonly occurring ticks in North America [24]. As in this study, experimental infestation studies typically involve assessments at 48 h post-treatment, and 48 h after subsequent weekly challenges. As tick transmission of some protozoal pathogens may not occur until up to 72 h post-infestation, the demonstration of lotilaner efficacy against *A. cajennense* (*s.l*.) at 48 h after challenge provides assurance that the risk of disease from a tick bite can be substantially reduced [25, 26]. For lotilaner, this was shown in an earlier study in which treatment successfully prevented the transmission of *Babesia canis* to dogs exposed to infected *D. reticulatus* ticks [27].

## Conclusions

This study demonstrates that lotilaner given orally at a minimum dose rate of 20 mg/kg is well tolerated, provides a rapid reduction of existing nymphal *A. cajennense* (*s.l*.) tick infestations, and provides a sustained residual protection for at least 30 days against infestation by *A. cajennense* (*s.l*.) ticks in dogs. This level of activity indicates that lotilaner can be a valuable tool to help prevent the transmission of tick-borne diseases to treated dogs [27].

## Abbreviations

GABA: γ-aminobutyric acid
Mc: mean number of live ticks on dogs in the untreated control group
Mt: mean number of live ticks on dogs in the treated group
na: not applicable
SD: standard deviation
WAAVP: World Association for the Advancement of Veterinary Parasitology
